# The Cisplatin-Derived Increase of Mitochondrial Reactive Oxygen Species Enhances the Effectiveness of Photodynamic Therapy via Transporter Regulation

**DOI:** 10.3390/cells8080918

**Published:** 2019-08-17

**Authors:** Hiromi Kurokawa, Hiromu Ito, Hirofumi Matsui

**Affiliations:** 1 Faculty of Medicine, University of Tsukuba, Ibaraki 305-8575, Japan; 2 Graduate School of Medical and Dental Sciences, Kagoshima University, Kagoshima 890-8544, Japan

**Keywords:** Photodynamic therapy, cisplatin, 5-aminolevulinic acid, peptide transporter 1, ATP-binding cassette member 2 of subfamily G

## Abstract

Photodynamic therapy (PDT) is a cancer treatment involving the generation of reactive oxygen species (ROS) by laser irradiation of porphyrins that accumulate in cancer tissues. 5-aminolevulinic acid (ALA), a porphyrin precursor, is often used as a photosensitizer. ALA is imported into cells via peptide transporter 1 (PEPT1), and porphyrin is exported via ATP-binding cassette member 2 of subfamily G (ABCG2). Thus, cancer cell-specific porphyrin accumulation involves regulation of both transporters to enhance the ALA-PDT effect. We reported previously that mitochondrial ROS (mitROS) upregulated PEPT1 expression and downregulated ABCG2 expression. Therefore, we propose that increasing mitROS production will enhance ALA-PDT cytotoxicity. Cisplatin is a chemotherapeutic drug that induces intracellular ROS generation. In this study, we investigated whether cisplatin-increased mitROS production in gastric cancer cell lines (RGK36 and RGK45) enhanced the cytotoxicity of ALA-PDT by regulation the expression of both PEPT1 and ABCG2. The results showed that cisplatin increased intracellular mitROS production in cancer but not normal cells (RGM1). PEPT1 was upregulated and ABCG2 downregulated in cancer cells treated with cisplatin. Moreover, intracellular porphyrin accumulation and ALA-PDT cytotoxicity increased. We conclude that cisplatin treatment increases the intracellular mitROS concentration and upregulates PEPT1 and downregulates ABCG2 expression.

## 1. Introduction

Photodynamic therapy (PDT) was developed as a modality for cancer treatment. It utilizes a combination of a low energy laser and photosensitizer [1]. There are two types of photosensitizers used in cancer therapy: a heme precursor such as 5-aminolevulinic acid (ALA), and compounds containing a porphyrin ring, including porphyrin and chlorine [2]. ALA is a porphyrin precursor that is synthesized from glycine and succinyl Co-A in mitochondria and is used as a photosensitizer in PDT [2,3]. ALA has several advantages compared to other photosensitizers. Specifically, it is rapidly cleared from tissues and the body within 48 h [4,5]. Moreover, ALA is not a fluorophore itself and never generates ROS without photodynamic excitation. Importantly, the porphyrins used for PDT tend to accumulate in cancer cells but not normal cells. Therefore, ALA-PDT causes less photosensitive dermatitis and retinal inflammation. Clinical trials for ALA-PDT are conducted worldwide [6], and more cancer-specific therapeutic effects are desired.

The porphyrin derivatives used in PDT generate singlet oxygen and other reactive oxygen species (ROS) by laser irradiation of the porphyrin accumulated in cancer tissues. Therefore, cancer cell-specific ALA uptake is important for efficient ALA-PDT [7,8]. Intracellular ALA influx is mediated by intestinal peptide transporter 1 (PEPT1) [9,10]. ATP-binding cassette member 2 of subfamily G (ABCG2) exports porphyrins across the plasma membrane to maintain intracellular porphyrin homeostasis [11]. Thus, achieving effective ALA-PDT requires the upregulation of PEPT1 expression and downregulation of ABCG2 expression. We reported previously that rat gastric epithelial cancer cells with a higher mitochondrial ROS (mitROS) concentration exhibited high PEPT1 expression [12]. We also reported that enhancing the intracellular mitROS concentration by hyperthermia resulted in downregulation of ABCG2 in cancer cells [13]. Based on these results, we hypothesize that an increase of the mitROS concentration can enhance the effects of ALA-PDT.

To increase mitROS production, we focused on cisplatin. Cisplatin is a platinum coordination compound that induces cytotoxicity by the formation of DNA adducts. These include DNA-protein cross-links, DNA monoadducts, and inter- and intra-strand DNA cross-links [14]. Cisplatin has been used for treating several human cancers such as gastric and esophageal cancers [15]. Cisplatin damages DNA in both the nuclear and mitochondrial compartments [16]. In mitochondria, the capacity of p53 to directly activate Bax to permeabilize this organelle permits an uninterrupted pathway leading from DNA damage to the mitochondrial release of cytochrome c, caspase activation, and apoptosis [17]. Even at cisplatin concentrations at which apoptosis does not occur, the intracellular ROS concentration increases [18]. In this study, we investigated whether low concentrations of cisplatin could accelerate mitROS production in cancer cells to enhance the cytotoxicity of ALA-PDT by regulating the expression of both PEPT1 and ABCG2.

## 2. Materials and Methods

### 2.1. Cell Culture

RGM1 rat gastric mucosal cells was purchased from RIKEN cell bank (Tsukuba, Japan). RGK1, the original cell of RGK36 and RGK45 cells were established by exposing 1-Methyl-3-nitro-1-nitrosoguanidine to RGM1. RGK36 and RGK45 were obtained from RGK1 by limiting dilution method and show the different character [13]. RGM1 was cultured in Dulbecco’s modified Eagles/F12 medium (Life Technologies Japan, Tokyo, Japan). RGK36 and RGK45 were cultured in Dulbecco’s modified Eagles/F12 medium without L-glutamine (Sigma-Aldrich Japan K.K., Tokyo, Japan). These culture media contained 10% heat-inactivated fetal bovine serum (Biowest, Kansas City, MO, USA) and 1% penicillin/streptomycin (Wako Pure Chemical Industries, Osaka, Japan). Cells were cultured in a 37 °C incubator in an atmosphere of 5% CO_2_ in air.

### 2.2. Cell Viability Assay

Cell viability was examined using Cell Counting Kit-8 (CCK-8) (Dojindo, Tokyo, Japan) according to the manufacturer’s protocol. RGK36, RGK45, and RGM1 cells were cultured on 96-well plates at 2 × 10^3^ cells/well and incubated overnight. The supernatant was aspirated, and the medium replaced. Cytotoxicity was determined by incubating cells at 37 °C for 24 h in the presence of 0, 1, 5, 10, and 20 μM cisplatin (Wako Pure Chemical Industries, Osaka, Japan). After incubation, cells were rinsed twice with phosphate-buffered saline (PBS), then incubated with 10% CCK-8 reagent. Absorbance at 450 nm was measured by a DTX880 multi-mode microplate reader (Beckman Coulter, Brea, CA, USA).

### 2.3. Electron Spin Resonance (ESR) Spectroscopy

ROS generation in cells was measured using ESR according to a previous study [19]. Cells were seeded on a glass cover slip (49 × 5 × 0.2 mm) and incubated until confluence. Then, cells were exposed to 10 μM cisplatin for 1 h. After treatment, cells were suspended in a respiration solution containing 5 mM succinate (Sigma-Aldrich Japan K.K., Tokyo, Japan), 5 mM glutamate (Sigma-Aldrich Japan K.K., Tokyo, Japan), 5 mM malate (Sigma-Aldrich Japan K.K., Tokyo, Japan), 5 mM NADH (Dojindo, Kumamoto, Japan), and a spin trapping agent (5.9% (v/v) DMPO (Labotec Co., Tokyo, Japan)). ESR spectra were recorded using a JEOL-TE X-band spectrometer (JEOL, Tokyo, Japan). All ESR spectra were obtained under the following conditions: 7.5 mT sweep width, 0.1 mT modulation width, 0.1 s time contrast, 335.5 mT center field, and 9.4 GHz frequency.

### 2.4. Measurement of Mitochondrial ROS

RGK36, RGK45, and RGM1 cells were incubated for 72 h in 96-well plates at 1 × 10^3^ cells/well. Cells were treated with 10 μM cisplatin for 1 h. After treatment, cells were incubated for 30 min in 5 μM MitoSOX (Life Technologies, Carlsbad, CA, USA) diluted with medium without phenol red. Cells were then rinsed with PBS and placed in fresh medium without phenol red. The fluorescence intensity of MitoSOX was measured by a Varioskan microplate reader (Thermo Fisher Scientific K.K., Kanagawa, Japan) at excitation and emission wavelengths of 510 and 580 nm, respectively.

### 2.5. Intracellular Porphyrin Accumulation after ALA Treatment

RGK36, RGK45, and RGM1 cells were cultured overnight in 12-well plates at 5 × 10^4^ cells/well. Cells were then incubated with 10 μM cisplatin for 1 h, rinsed twice with PBS, then incubated for an additional 24 h. The cells were then incubated with 1 mM ALA (Cosmo Bio, Tokyo, Japan) for 6 h, rinsed with PBS, and lysed in 100 μL of RIPA buffer. Cell lysates were transferred to a 96-well plate and fluorescence intensity of the hematoporphyrin derivative was measured using a Varioskan microplate reader with excitation and emission wavelengths of 415 and 625 nm, respectively.

### 2.6. Cellular Uptake of ALA

RGK36, RGK45, and RGM1 cells were cultured as described in Section 2.5. After the 24 h incubation, cells were incubated with 1 μM radiolabeled [4-^14^C]-ALA (American Radiolabeled Chemicals, St. Louis, MO, USA) for 24 h. The cells were rinsed with PBS and lysed in 500 μL radioimmunoprecipitation assay buffer. Cell lysates were transferred to vials containing liquid scintillation fluid (Pico-Fluor 40) and radiation counts were determined in a liquid scintillation counter (LSC-7200, Hitachi Aloka Medical, Tokyo, Japan).

### 2.7. Cell Viability Assay after PDT

RGK36, RGK45, and RGM1 cells were incubated overnight in 96-well plates at 1 × 10^3^ cells/well, then incubated with 10 μM cisplatin for 1 h and rinsed twice with PBS. Thereafter, the cells were incubated for 24 h. After treatment, cells were incubated with 1 mM ALA for 24 h, rinsed twice with PBS, and then placed in fresh medium without phenol red. Cells were irradiated by excimer dye laser light (630 nm, 0.5 J/cm2) using an EDL-1 laser diode driver (Hamamatsu Photonics K.K., Hamamatsu, Japan). After irradiation, cells were incubated for 24 h. The medium was replaced with fresh medium containing 10% CCK-8 reagent and the cells were further incubated. Absorbance at 450 nm was measured on a DTX880 multi-mode microplate reader.

### 2.8. Statistical Analysis

Data are expressed as the means ± SD and were assessed by an analysis of variance. Individual groups were compared by Tukey’s post-hoc or Student’s t-test with *p* < 0.05 considered statistically significant.

## 3. Results

### 3.1. Cisplatin-Induced Cytotoxicity in RGK36, RGK45, and RGM1 Cells

**Figure 1:**
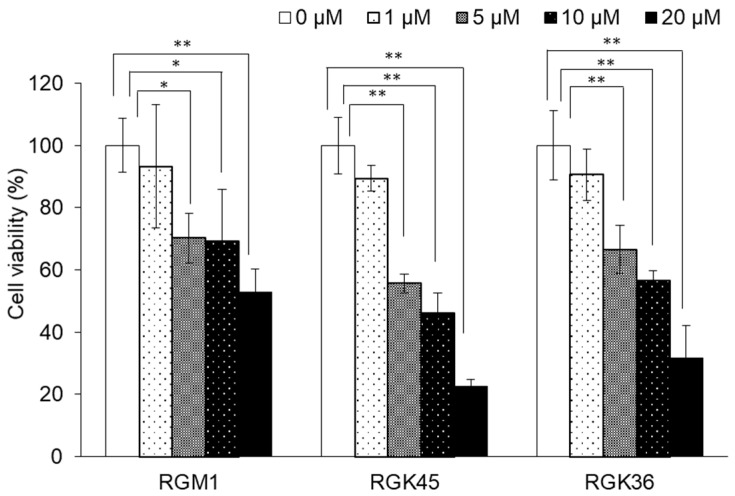
Cytotoxicity of cisplatin. Cell viability in all three cell lines decreased significantly in a concentration-dependent manner. Data are expressed as means ± SD (n = 4). * *p* < 0.05, ** *p* < 0.01.

The cytotoxicity of cisplatin to RGK36 (cancer), RGK45 (cancer), and RGM1 (normal) cells was evaluated by the CCK-8 assay. Cell viability decreased significantly in all three cell lines in a concentration-dependent manner (5, 10, and 20 μM) 24 h after cisplatin exposure (Figure 1). The cytotoxicity of cisplatin was higher in cancer than in normal cells at all concentrations.

### 3.2. Intracellular ROS Production 

**Figure 2:**
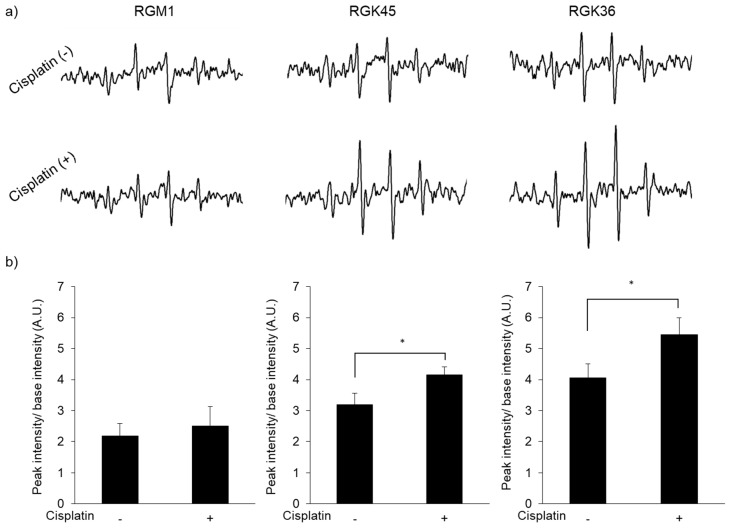
Intracellular reactive oxygen species (ROS) production after treatment with or without cisplatin (10μM). (**a**) The electron spin resonance (ESR) spectra. (**b**) Relative ESR intensity. Intracellular ROS concentration in RGK36 and RGK45 were increased after cisplatin treatment, while RGM1 cells were not. Data are expressed as means ± SD (n = 3). * *p* < 0.05, ** *p* < 0.01.

**Figure 3:**
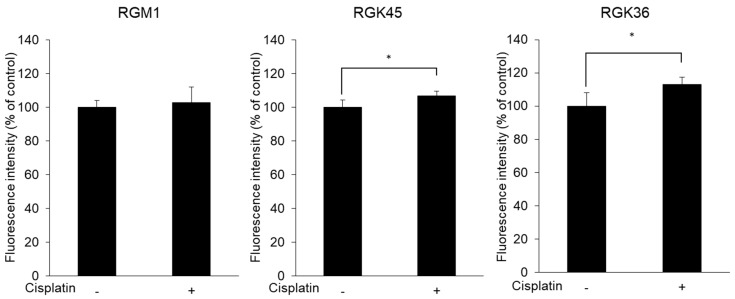
Fluorescence intensity of MitoSOX. MitoSOX intensity was increased after cisplatin treatment (10 μM) in RGK36 and RGK45. Ex. 510 nm and Em. 580 nm. Data are expressed as means ± SD (n = 4). * *p* < 0.05.

Cisplatin exposure increased intracellular ROS production. The ESR signal intensity of DMPO in RGK45 and RGK36 cells was enhanced by 10 μM cisplatin, while in RGM1 cells the signal did not change (Figure 2a,b). Intracellular ROS production was also detected with MitoSOX, a mitochondrial superoxide indicator. The fluorescence intensity increased in RGK36 and RGK45 cells treated with cisplatin, while it was unchanged in RGM1 cells (Figure 3). These results demonstrate increased intracellular ROS production by cisplatin in cancer cells but not normal cells. 

### 3.3. PEPT1 and ABCG2 Expression

**Figure 4:**
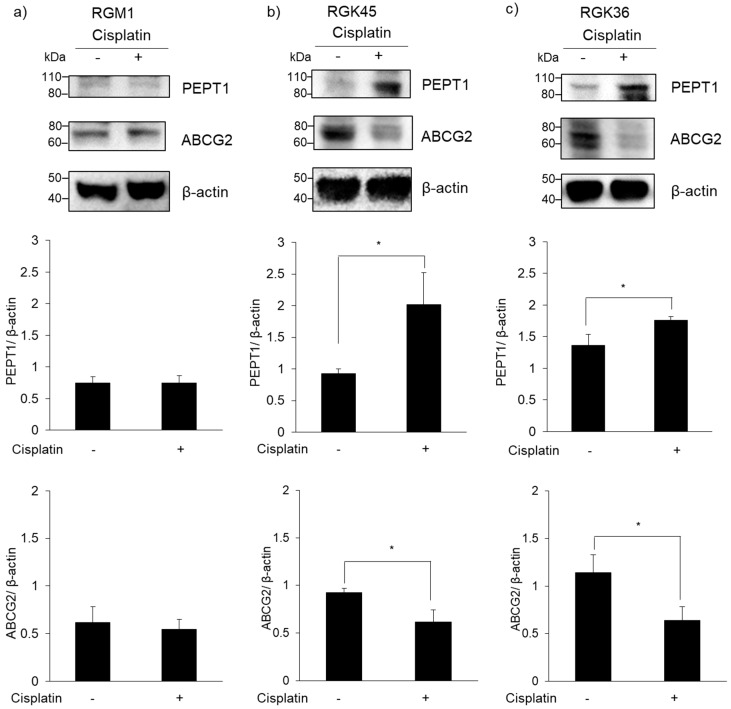
Expression of peptide transporter 1 (PEPT1) and ABCG2. (**a**) RGM1, (**b**) RGK45 and (**c**) RGK36. PEPT1 and ABCG2 expressions in RGK45 and RGK36 cells were changed by cisplatin treatment (10 μM). Data are expressed as the mean ± SD (n = 3). * *p* < 0.05.

Intracellular levels of PEPT1 and ABCG2 were examined by western blotting. PEPT1 expression was upregulated in RGK36 and RGK45 cells by cisplatin exposure, while ABCG2 expression was downregulated (Figure 4). The expression of these transporters was unchanged by cisplatin exposure in RGM1 cells. 

### 3.4. Intracellular Porphyrin Accumulation after ALA Addition

**Figure 5:**
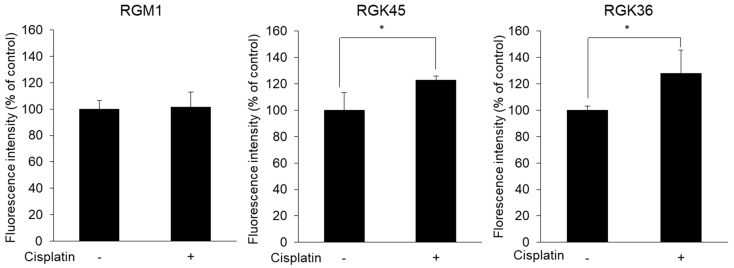
Porphyrin accumulation in each cell after 5-aminolevulinic acid (ALA) exposure (1 mM). Porphyrin fluorescence intensity derived ALA increased by cisplatin treatment (10 μM) in RGK45 and RGK36 cells. Data are expressed as means ± SD (n = 4). * *p* < 0.05.

Cells were exposed to 10 μM cisplatin for 1 h. Twenty-four hours after incubation, cells were treated with 1 mM ALA for 24 h, then intracellular porphyrin fluorescence was measured. The fluorescence intensity of porphyrin increased significantly to 123 and 128% of control cells in RGK45 and RGK36 cells, respectively, whereas it was unchanged in RGM1 cells (Figure 5). 

### 3.5. Cancer Cell-Specific Uptake of ALA

**Figure 6:**
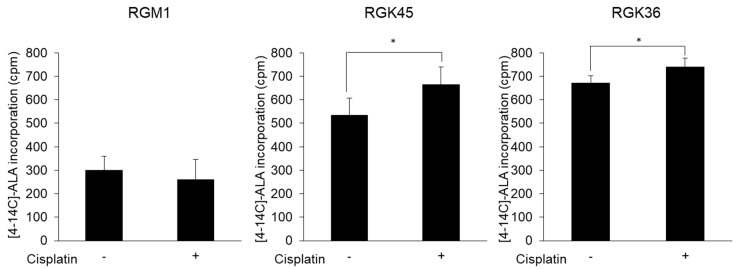
Cellular uptake of 14C-labeled ALA. ALA uptake increased specifically in cancer cells by cisplatin treatment (10 μM). Data are expressed as means ± SD (n = 4). * *p* < 0.05.

Intracellular ALA uptake was examined using radio-labelled ALA. Intracellular ALA levels were increased by cisplatin exposure in RGK36 and RGK45 but unchanged in RGM1 cells (Figure 6). These data indicate that ALA uptake is higher in cancer than in normal cells.

### 3.6. Cell Viability after PDT

**Figure 7:**
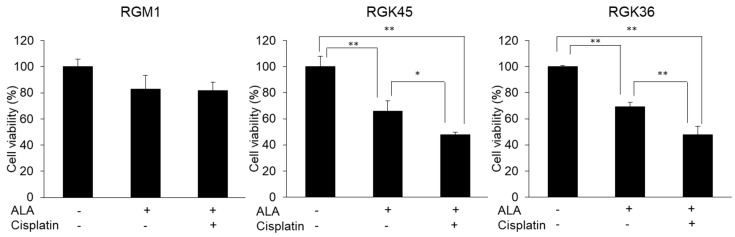
The effect of combination therapy with both cisplatin and photodynamic therapy (PDT). Cells were treated by 10μM cisplatin, then exposed 1 mM ALA. Cytotoxicity of PDT enhanced by cisplatin treatment in both RGK45 and RGK36. Data are expressed as means ± SD (n = 4). * *p* < 0.05, ** *p* < 0.01.

The ALA-PDT effect was measured using the CCK-8 assay. Cytotoxicity by ALA-PDT in RGM1 cells was minimal with or without cisplatin treatment (Figure 7). ALA-PDT induced cancer cell-specific cytotoxicity as shown by the significant decrease in viability of RGK45 and RGK36 cells to 66 and 69% of control, respectively. Moreover, the ALA-PDT effect was enhanced by cisplatin treatment and viability in both cell lines was significantly decreased to 48% of control.

## 4. Discussion

In this study, we demonstrated that intracellular mitROS production was enhanced by low concentrations of cisplatin. We also showed that PEPT1 expression was upregulated and ABCG2 expression downregulated by cisplatin. These results suggested that cisplatin would enhance cancer cell-specific cytotoxicity by ALA-PDT. 

The purpose of cisplatin treatment is to increase the production of mitROS, not to induce cell death directly. Based on the results of the cell viability assay, we investigated mitROS production in RGK36 and RGK45 cells treated with 10 μM cisplatin for 1 h. Intracellular ROS production was also measured with ESR, which showed significant increases in RGK45 (*p* = 0.02) and RGK36 (*p* = 0.03) cells. However, the ESR signal intensity did not increase in RGM1 cells. To determine the species of intracellular ROS produced by cisplatin exposure, we used MitoSOX, which is a superoxide indicator in living cells [20,21]. The fluorescence intensity of MitoSOX was increased in RGK45 (*p* = 0.04) and RGK36 (*p* = 0.03) cells. Thus, cisplatin treatment induced cancer cell-specific mitROS production.

Porphyrin is synthesized from ALA as follows: 1) ALA, which is a precursor of porphyrin, is synthesized from glycine and succinyl Co-A; 2) porphobilinogen is generated from ALA and then converted into hydroxymethylbilane; 3) hydroxymethylbilane is converted into uroporphyrinogen III, coproporphyrinogen III, and protoporphyrinogen IX; and 4) protoporphyrin IX is synthesized by protoporphyrinogen oxidase [22]. Thus, treatment with ALA increases intracellular porphyrin accumulation that is then utilized to enhance the efficacy of PDT and photodynamic diagnosis. 

Cancer cell-specific ALA accumulation is very important for ALA-PDT or ALA-photodynamic diagnosis. The expression of PEPT1, one of the ALA transporters [9,10], is higher in cells with high mitROS production than cells with low mitROS production [12]. MitROS also regulate HCP-1 expression [23]. HCP-1 is known as a heme carrier transporter and also transport the porphyrins synthesized from ALA [23]. We have shown that the intracellular mitROS concentration is increased by indomethacin treatment or hyperthermia, and HCP-1 expression is subsequently enhanced [13,24]. Since PEPT1 expression also depends on the amount of mitROS production, we considered that increase of the exogenous mitROS production can upregulate the PEPT1 expression and consequently enhance the effect of ALA-PDT. We also reported that increased mitROS levels downregulated ABCG2 expression [13]. In this study, we investigated whether transporter expression can be regulated by a method that is easier to use clinically. Cisplatin is a typical anticancer drug used in various cancer chemotherapies [14]. In the current study, low concentrations of cisplatin increased mitROS production. Thus, we hypothesized that cisplatin treatment can upregulate PEPT1 expression and downregulate ABCG2 expression. 

The expression of neither transporter was altered in RGM1 cells treated with cisplatin. However, PEPT1 expression increased, while ABCG2 expression decreased in both RGK45 and RGK36 cells treated with cisplatin (Figure 4). Cisplatin treatment significantly enhanced intracellular fluorescence intensity and cellular ALA uptake in both cancer cell lines (Figure 5 and Figure 6). From these results, we proposed that cisplatin treatment increased the intracellular mitROS concentration and ALA uptake by regulating transporter expression specifically in cancer cells. We have previously found that HIF-1α stabilization upregulates HCP-1 expression. PEPT1 is a SLC family similar to HCP-1 and we consider that stabilization of HIF-1α enhanced PEPT1 expression. Details of the regulation of ABC transporter are currently under investigation, however we hypothesize that a decrease in ABCG2 expression is also regulated by HIF-1α stabilization.

PDT is a cancer therapy that takes advantage of cancer cell-specific porphyrin accumulation. In the current study, ALA-PDT showed no effect on the viability of RGM1 cells. Moreover, these normal epithelial cells were not substantially injured by combined treatment with ALA-PDT and cisplatin. In contrast, ALA-PDT alone induced severe cytotoxicity in both cancer cell lines. Furthermore, combined therapy with ALA-PDT and 10 μM cisplatin significantly increased cytotoxicity. Cisplatin exposure at this protocol was 10 microM for 1 h which does not show cytotoxicity. Thus, it was shown that treatment with a concentration of non-cytotoxic cisplatin alone can enhance the ALA-PDT effect. Previous reports also indicated that this combination enhanced cytotoxicity. For example, Ahn et al. reported that the combination ALA-PDT and cisplatin synergistically enhanced the cytotoxicity of PDT both in vitro and in vivo [25]. Wei et al. also reported that cytotoxicity was enhanced by the combination ALA-PDT and cisplatin [26]. They further reported that the combination induced apoptosis. Yu et al. reported that the cytotoxic effect of cisplatin was significantly increased with the addition of ALA-PDT [27]. These studies show that cisplatin alone is a concentration that causes mild cell injury, however a combination with PDT can induce a synergistic cytotoxic effect. On the other hand, we found that despite the concentration, cytotoxicity is not caused by cisplatin alone, the PDT effect can be enhanced by regulating the expression of transporter by cisplatin. We achieved the enhancement of ALA-PDT effect by low concentration cisplatin treatment. This cisplatin concentration did not affect normal cells. Cisplatin treatment increased mitROS production and regulated the expressions of transporters in only cancer cells. Thus, we suggest that combination therapy with ALA-PDT and low concentration of cisplatin can be a highly selective cancer treatment with few side effects.

## 5. Conclusions

The results of this study showed that low concentrations of cisplatin increased the intracellular mitROS concentration, and upregulated PEPT1 expression and downregulated ABCG2 expression. These changes appear to explain why the combination of cisplatin and ALA-PDT exerts a synergistic effect on cancer but not normal cells. Cisplatin is one of the most used anti-cancer drugs in the clinic and is used in various cancers. The ability to enhance the PDT effect with such casual drugs is expected to have a rapid clinical application.
